# Psychophysiological Benefits of Real-Time Heart Rate Feedback in Physical Education

**DOI:** 10.3389/fpsyg.2021.651065

**Published:** 2021-03-16

**Authors:** Tino Stöckel, Robert Grimm

**Affiliations:** Sport and Exercise Psychology Unit, Department of Sport Science, University of Rostock, Rostock, Germany

**Keywords:** self-determination theory, heart rate monitoring, motivation, biofeedback, school

## Abstract

School physical education (PE) has the potential to contribute to public-health promotion and well-being, but oftentimes students' lack of motivation toward PE or physical activity in general, especially during adolescence, diminishes, or eradicates the positive effects associated with PE. Therefore, practical approaches are required that help teachers to increase or awake students intrinsic motivation toward PE, for which self-determination theory may provide the conceptual framework. In that regard, the purpose of the present study was to examine whether the use of real-time, heart rate feedback (as a method to support students' need for autonomy and competence) during regular PE lessons has the potential to increase students' autonomous motivation and physical effort. To achieve this, we had forty healthy adolescents between 16 and 17 years of age run for 30 min either with (experimental group, EG) or without (control group, CG) real-time, individualized heart rate feedback during a regular PE class and compared physical and perceived exertion as well as joy of running between the two groups. Participants were randomly assigned to the groups. Our data revealed that participants in the EG enjoyed running more than participants in the CG (joy of running was 3.20 in the EG vs. 2.63 in the CG, *p* = 0.03) despite a higher physical (163 to 178 in EG vs. 141 to 156 beats per minute in the CG, *p* < 0.001) and perceived exertion (rating of perceived exertion of 13.22 in the EG vs. 10.59 in the CG, *p* = 0.02). That means, running with real-time, individualized heart rate feedback apparently increased participants' motivation to run and to enjoy running at higher levels of exertion. In that regard, real-time, individualized activity feedback should be implemented in regular PE classes systematically and repeatedly to create a controllable and attainable situation that allows students to actively adjust their own behavior to achieve appealing and realistic goals.

## Introduction

Physical activity has long been known as a major, independent risk factor of individual and public health and well-being (World Health Organization, 2010; Hallal et al., 2012; Lee et al., 2012). As regular physical activity during childhood benefits all aspects of child development (Andersen et al., 2006; Reiner et al., 2013), it is recommended that children engage in 60 min of moderate (MPA) to vigorous aerobic physical activity (VPA; i.e., fast walking and running associated with elevated heart rates) every day (Strong et al., 2005; Janssen and LeBlanc, 2010; World Health Organization, 2010). Depending on the criteria and model used (Epstein et al., 2001; Armstrong and Welsman, 2006; Brown et al., 2006; McManus et al., 2008; American College of Sports Medicine, 2014; Eckard et al., 2019), MPA is typically defined with heart rates above 64–70% of maximum heart rate, or heart rates ranging between 140 and 160 beats per minute (bpm); and VPA with heart rates above 76–85% of maximum heart rate or with heart rates above 160 bpm. Field observations, however, indicated that not even half of the children and adolescents comply with the recommended levels of physical activity (Ekelund et al., 2011; Van Hecke et al., 2016). Actual daily engagement in moderate to vigorous physical activity in school-aged children is (with about 14–20 min on average) well below the recommended 60 min per day (Biddle and Goudas, 1996; Sallis et al., 2000; McManus et al., 2008). Making things worse, it has been reported that moderate and vigorous physical activity declines dramatically with increasing age during adolescence (Armstrong et al., 2000; Parish and Treasure, 2003; McManus et al., 2008; Knuth and Hallal, 2009; Van Hecke et al., 2016). Hallal et al. (2012) reported that worldwide four out of five adolescents do not meet the physical activity recommendations.

Physical education (PE) in school may have the potential to contribute to public-health promotion (Fox and Harris, 2003; Standage and Gillison, 2007; Singh et al., 2012; Burns et al., 2017) as it is mandatory for almost all children and adolescents in the world. However, the recommendations for daily physical activity are usually not met by PE in school alone as it only covers 2–3 days of a week of the recommended daily 60 min of moderate to vigorous physical activity (given the intensity is high enough; cf. Stratton, 1996; Fröberg et al., 2017). Therefore, it is required that children engage in extracurricular physical activity to make up for the lack. PE appears to be the perfect place to build up children's motivation to engage in extracurricular physical activities as a central aim of PE in school is to encourage students to take on a physically active and healthy lifestyle. It is generally agreed upon that this can be achieved by growing children's understanding of the benefits of an active and healthy lifestyle and by letting them experience that physical activity can bring joy and inherent satisfaction (Ferrer-Caja and Weiss, 2000; Vallerand, 2007; Ryan and Deci, 2017). In particular, enjoyment of PE has been shown to have a considerable positive impact on children's extracurricular physical activity (Dishman et al., 2005; Cairney et al., 2007; Cox et al., 2008).

The Self-determination theory (SDT; Deci and Ryan, 2000; Ryan and Deci, 2000, 2007, 2017) provides a conceptual framework on how autonomous forms of motivation, which are typically accompanied by the feeling of enjoyment and inherent satisfaction, can be achieved. It describes different modes of regulation with external regulation being the least and intrinsic motivation being the most self-determined and autonomous forms of regulation (Ryan and Deci, 2000; Deci and Ryan, 2002). With regard to PE (or school in general), children and adolescents often act for external reasons (Ntoumanis, 2001; Ntoumanis et al., 2004), which are usually associated with the expectation of reward and/or punishment (e.g., external pressure by parents of getting good or avoiding bad marks) and the avoidance of guilt associated with not partaking or underperforming (e.g., internal pressure based on perceived expectations of classmates). However, acting for internal reasons (i.e., intrinsic, identified, and introjected regulation) is typically associated with a higher engagement and a stronger feeling of enjoyment, interest and inherent satisfaction (e.g., partaking in PE because being physical active or a specific behavior itself has personal value or brings the student joy, not just the outcome) as compared to acting for external reasons (i.e., lower autonomous motivation) (Ryan and Deci, 2000, 2017; Spray et al., 2006; Vallerand, 2007; Bice et al., 2016).

SDT proposes that autonomous motivation can be achieved by promoting the satisfaction of three innate psychological needs: competence, autonomy and relatedness (Deci and Ryan, 2002; Standage and Gillison, 2007; Ryan and Deci, 2017). *Competence* describes one's feeling to be able (i.e., to have what it takes) to effectively adjust own behavior to achieve desired outcomes. Several studies have come to the conclusion that perceived competence might be the most important predictor for intrinsic motivation toward and enjoyment of PE (Standage et al., 2005; Hashim et al., 2008; Taylor et al., 2010; Gråstén et al., 2012; Ryan and Deci, 2017). *Autonomy* describes the need to self-endorse activities and the feeling to be able to handle situations on one's own. It has been shown that autonomy support provided by the teacher by actively supporting choice, initiation and understanding enhances motivation toward PE and extracurricular physical activity (Hagger et al., 2003; Standage et al., 2003, 2006; Reeve et al., 2004; Alderman et al., 2006). *Relatedness* describes the need to belong, to feel connected, accepted, and close to significant others. Relatedness appears not as important for enhancing and maintaining autonomous forms of motivation as autonomy and competence (Deci and Ryan, 2000; Standage and Gillison, 2007), but there is evidence to suggest that it supports maintaining motivated when acting for external reasons (Standage and Gillison, 2007). Satisfaction of the three psychological needs, especially competence and autonomy, in PE has been shown to be positively associated to autonomous forms of motivation toward PE (Standage et al., 2003, 2006; Ntoumanis, 2005; Standage and Gillison, 2007). Moreover, autonomous motivation toward PE has been positively associated to general self-esteem and psychological well-being (Hein and Hagger, 2007; Standage and Gillison, 2007) supporting the idea that (motivational processes within) PE has the potential to impact on self-perception and a healthy active lifestyle.

### The Present Study

Especially during adolescence, many students are only physically active in PE for external reasons (i.e., because they have to or to avoid bad marks) or are not being motivated to be physically active at all (Ntoumanis, 2001; Ntoumanis et al., 2004) resulting in low levels of physical activity during PE classes (Knuth and Hallal, 2009). That completely undermines the central aim of PE to encourage students to take on a physically active and healthy lifestyle and the recommendation to engage in at least 60 min per day of moderate to vigorous physical activity. Therefore, the purpose of the present study was to explore the effect of real-time activity feedback on adolescents' experience of intrinsic satisfaction of being physically active during PE (i.e., enjoying the activity due to the feeling of improvement and accomplishment; cf. Wankel and Kreisel, 1985; Vallerand, 2007; Bice et al., 2016). More specifically, we examined whether the use of immediate heart rate feedback during endurance training (i.e., running) in PE has the potential to increase students' joy of running and motivation to run as they may feel more in control of the situation and their own performance when being provided with immediate and individualized heart rate feedback.

During regular PE lessons, we asked forty adolescents between 16 and 17 years of age to run for 30 min at their maximum pace that would allow them to run for 30 min without any breaks. Participants were evenly and randomly assigned to either an experimental (EG) or a control group (CG). All participants wore heart rate monitors while running and were asked to report their perceived level of exertion and on whether they enjoyed running after the 30-min run. The groups only differed in the information provided about heart rate monitoring before running and the feedback given during running. Participants in the CG were just told that the heart rate monitor measures and records their hearts' bpm during the 30-min run. Participants in the EG were given additional information on what that means (i.e., information on how hard they push themselves while exercising) and how the heart rate feedback can be used during running (i.e., information on how to run in an optimal healthy heart rate zone to finish the 30-min run without breaks and to get better at running). Moreover, during running participants in the EG received real-time heart rate feedback (i.e., heart rate, percentage of their maximum heart rate and heart rate zone) displayed on a projector screen visible from all sides of the gym.

We hypothesized that participants in the EG enjoy running more than participants in the CG, and (willingly) push themselves harder while exercising. Based on previous work, the individualized activity feedback (i.e., based on each participant's level of exercise) in the EG should help participants to feel more competent (Ntoumanis, 2001; Alderman et al., 2006). Moreover, the possibility to actively adjust their running speed based on the heart rate feedback at all times (i.e., they had control over their running speed and exhaustion) should support the need for autonomy (i.e., reinforcing behavior by self-evaluation; self-endorsed adjustments to achieve goals; controllability of the situation) in the EG. Consequently, the satisfaction of the basic psychological needs for competence and autonomy by providing real-time, individualized heart rate feedback should result in an increase in autonomous motivation toward running (i.e., enjoy running) (Ntoumanis, 2001; Standage et al., 2005; Gråstén et al., 2012; Bice et al., 2016; Nation-Grainger, 2017).

## Methods

### Participants

Forty healthy male adolescents (age range = 16–17 years, mean age = 16.3 ± 0.5 years) from a 10th grade volunteered in this study, and were randomly allocated to an experimental group (EG) and a control group (CG) before any testing. On average, participants in EG and CG engaged in 4.6 (± 3.5) and 3.5 (± 2.6) h of recreational physical activity per week respectively, indicating that the sport engagement was similar between both groups (*p* = 0.30). As body-mass index (BMI) was higher in the CG (22.9 ± 4.5) than in the EG (19.5 ± 2.7) (*p* = 0.01), BMI was used as a co-variate in all analyses of exertion in the present study. All participants were free from any known musculoskeletal, neuromuscular, neurological, and/or mental disorders that may have had an impact on test performance, and had normal or corrected to normal vision. The study was approved by the local institutional review board and school authorities, and conformed to the declaration of Helsinki. Prior to participation, written informed consent was obtained from the parents of all participants.

### Procedure

All participants completed two 30-min runs (separated by 1 week) in groups of 10 within their respective groups (i.e., EG and CG) during regular physical education lessons in a standard gym: The first running session was used for familiarization (i.e., wearing the heart rate monitor during running) and to determine the target heart rate zone for the experimental group. The second running session was used to test for the effect of heart rate feedback on (perceived) exertion and motivation by providing real time heart rate feedback in the experimental group, but not in the control group. All sessions were run by an instructor who was asked to limit interaction with participants to a minimum.

**Figure 1:**
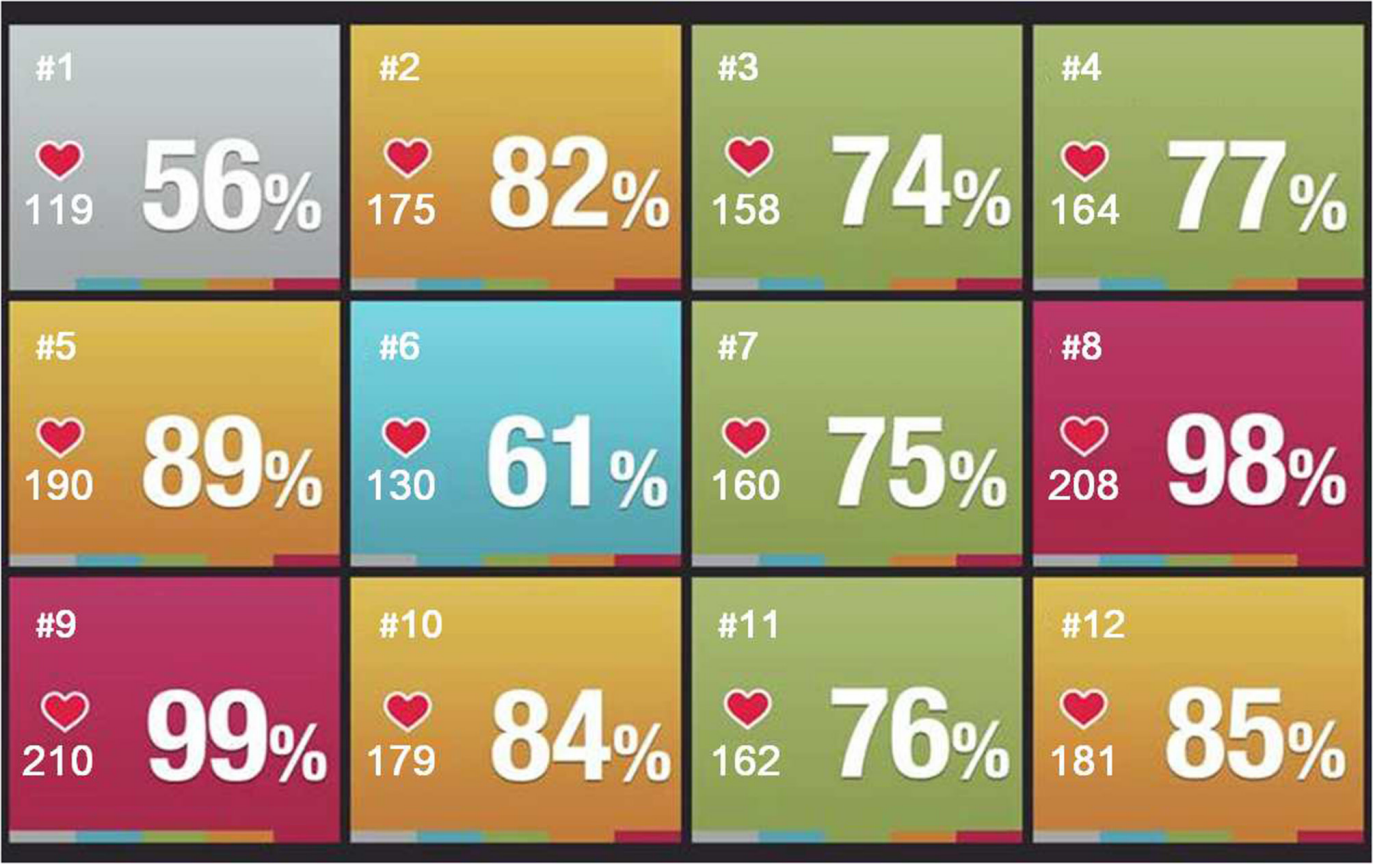
Heart-rate feedback as provided on the projector screen during running. Displayed are the number of the participant in the upper left corner, the real-time heart-rate in beats per minute in the lower left corner under a heart symbol, the respective percentage of their maximum heart-rate emphasized on the right side and the respective heart rate zone indicated by the background color. In the present example all information were provided for 12 participants at a time with participants being identified by their participant number.

Before any running, participants were asked (1) to report on whether they were looking forward to run or not (i.e., motivation), and (2) to put on a chest strap heart rate monitor (Polar H10). The experimenter always checked the correct position of the heart rate monitor and connected the respective heart rate monitor with the Polar Club App, which allowed real-time data recording and processing. Then, participants were asked to run for 30 min around a marked area (10 m × 20 m rectangle) in the maximum speed that would still allow them to run for 30 min without any breaks. After completing the 30-min run, participants were asked to report their perceived exertion (Borg's scale of perceived exertion), and on whether they enjoyed running (i.e., joy and motivation). EG and CG only differed in the information provided about heart rate monitoring before running and the feedback given during running. While participants in the CG were just told that the heart rate monitor measures and records their heart beats per minute (bpm) during the 30-min run, participants in the EG were given additional information on what that means (i.e., information on how hard they push themselves while exercising) and how the heart rate feedback can be used during running (i.e., information on how to run in an optimal healthy heart rate zone to finish the 30-min run without breaks and to get better at running). Moreover, during running participants in the EG received real-time heart rate feedback (i.e., heart rate in bpm, percentage of their maximum heart rate, and heart rate zone) displayed on a projector screen (120 inches) visible from all sides of the gym. Participants in the EG were instructed to always keep their heart rate between 80 and 90% (162/163 to 183/184 bpm; i.e., vigorous physical activity, American College of Sports Medicine, 2014) of the maximum heart rate (simplified as 220 bpm minus age; Fox et al., 1971), which was indicated by a yellow background color and the respective percentage on the projector screen (see Figure 1).

### Measures

#### Heart Rate Monitoring

Participants' heart rate data was collected using 10 Polar® H10 heart rate sensors attached to an adjustable chest strap with embedded electrodes to measure the heart's electrical signals. Previous studies provided evidence that chest strap monitors like the Polar H10 are (almost) as accurate as the current gold standard electrocardiography to obtain heart rates in sport settings (cf. Gilgen-Ammann et al., 2019). All data were transferred in real time via Bluetooth (with a maximum signal radius of about 35 m) to a 9.7″ Apple IPad, on which all data was stored and further processed using the Polar® Club App. To be able to match the simultaneously captured heart rate data of all participants within the EG and CG group to single subjects, we created individual accounts for all participants in the Polar® Club App and connected each participant's heart rate monitor with his or her account before any running started. Mean heart rate (in bpm) averaged over 30 min and time (in percent) in the five Polar® heart rate zones above 50% (i.e., 50–60% indicates very light, 60–70% light, 70–80% moderate, 80–90% intense, and 90–100% maximum intensity/exertion) were used as measures of physical exertion (i.e., how hard they push themselves during running). Following the guidelines of the American College of Sports Medicine (American College of Sports Medicine, 2014), moderate physical activity is associated with heart rates above 64% of maximum heart rate and vigorous physical activity with heart rates above 76% of maximum heart rate.

#### Perceived Exertion

Perceived exertion was assessed using Borg's rating of perceived exertion (RPE; Borg, 1985, 1998), a valid measure of exercise intensity (Chen et al., 2002) ranging from “no exertion” (score of 6) over “light exertion” (score of 11) and “hard exertion” (score of 15) to “maximum exertion” (score of 20). Immediately after running, all participants were handed the Borg's RPE scale showing scores, colors, smileys, and descriptions for each level of exertion (see Supplementary Material), and were asked to report their perceived level of exertion by making a cross at the respective number/description. Following the guidelines of the American College of Sports Medicine (American College of Sports Medicine, 2014), moderate physical activity is associated with RPE's of 12 to 13 and vigorous physical activity with RPE's of 14–17.

#### Motivation

Participants' motivation was assessed using short questionnaires before and after running. Before running, participants were asked to indicate on a 5-point Likert scale whether they are looking forward to the upcoming 30-min run (i.e., agree) or not (i.e., disagree). After running, participants were asked to indicate on the same 5-point Likert scale whether they enjoyed running or not. All possible responses were accompanied by sad (disagree), neutral (neither agree nor disagree), and happy (agree) smileys (see Supplementary Material) to make it easier for the participants to choose from the five possible responses (e.g., based on their own mood with regard to the upcoming or just completed run). The anticipation before running and the joy of running were used as a measure of motivation (Wankel and Kreisel, 1985; cf. Bice et al., 2016). A change in score from before to after the run will be interpreted as a change in motivation due to the running session itself.

After all running was completed, participants were also asked to report on the 5-point Likert scale explained above whether they enjoy running in general and whether they enjoyed running with the heart rate monitor. Responses to the first question were used to control data for participants' general running motivation and responses to the second question were used as direct feedback on whether participants liked the implementation of this technical gadget.

### Data Analysis

Preliminary analyses were conducted on all relevant measures to check for normality, sphericity (Mauchly test) and outliers, with no serious violations noted. In order to study the effect of enriched heart rate feedback on participants' exertion we ran separate analyses of variances (ANOVA) for physical (bpm averaged over 30 min) and perceived exertion (Borg's RPE) with group (EG vs. CG) as between-subject factor and controlled for participants' BMI. To compare experimental and control groups' time (in percent) spend in the five Polar® heart rate zones, we ran a group (EG vs. CG) × heart rate zone (1 through 5) ANOVA. Additionally we ran an ANOVA comparing the joy of running (controlled for the anticipation to run) between groups (EG vs. CG) to study the effect of enriched heart rate feedback on participants' motivation. Data are reported as mean (M) and 95% confidence interval of the mean (95% CI), as well as mean difference (MD) along with 95% CI. Partial eta-squared (η_*p*_^2^) and adjusted Cohens *d* (due to the rather small sample size) based on Ezekiel's correction formula (Ezekiel, 1930; Ivarsson et al., 2013; Schweizer and Furley, 2016) along with 95% CI of the effect size are reported as measures of effect size. The level of significance was set at *p* ≤ 0.05. All analyses were performed using SPSS statistical package 25.0.

## Results

### Physical Exertion

**Figure 2:**
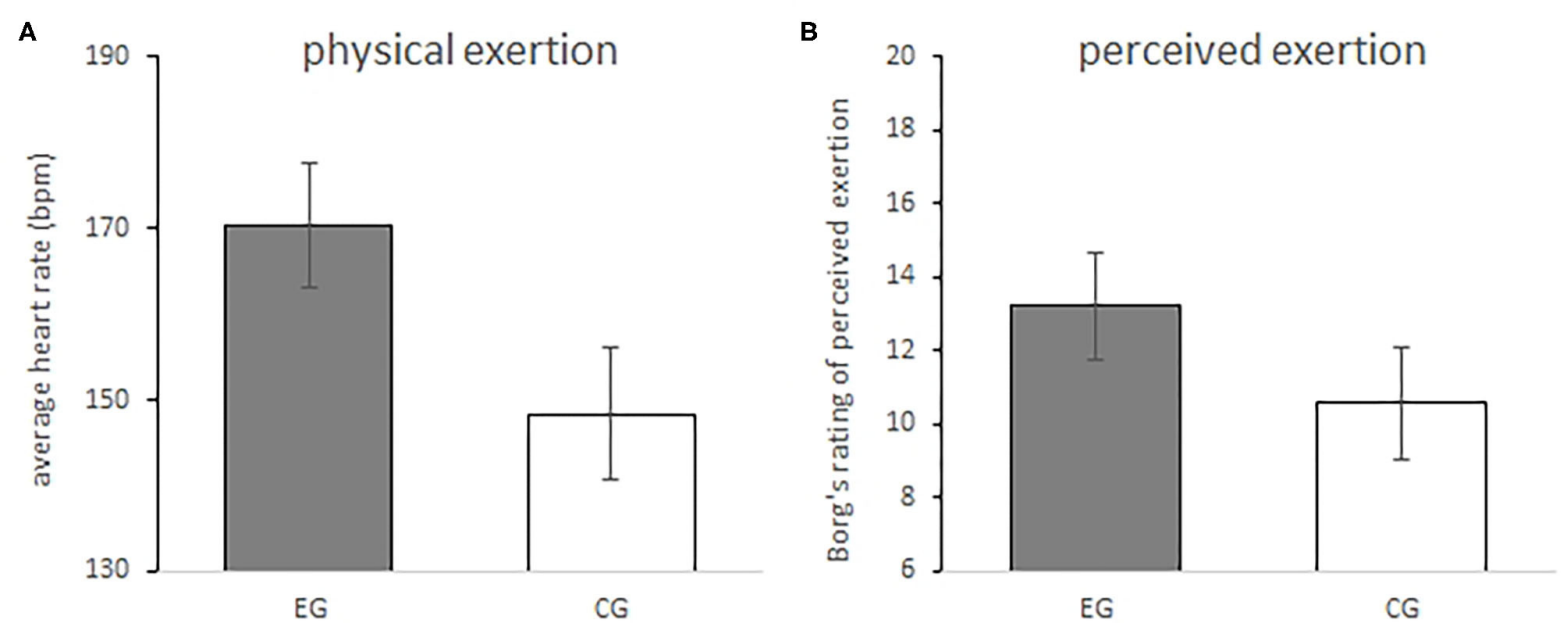
Physical **(A)** and perceived exertion **(B)** during the 30 min run in comparison between the experimental (EG, gray bar) and the control group (CG, white bar). Actual physical exertion during running (i.e., averaged over the 30 min run) was measured using heart rate monitors, with higher beats per minute (bpm) indicating higher physical exertion. For perceived exertion students were asked to self-report how hard they had to push during running using Borg's rating of perceived exertion (RPE). Higher RPEs indicate higher exertion. Error bars indicate 95% CI.

Raw means and standard deviations for all measures of physical and perceived exertion as well as anticipation and joy of running are displayed in Table 1. Data analysis revealed a significant difference between groups, *F*_(1,35)_ = 15.89, *p* < 0.001, η_*p*_^2^ = 0.31, with a higher physical exertion averaged over the 30 min of running in the EG (*M* = 170.34 bpm, *95% CI* = [163.11, 177.56]) as compared to the CG (*M* = 148.41 bpm, *95% CI* = [140.74, 156.08]) (adjusted Cohen's *d* = 1.27, *95% CI* = [0.54, 2.14]). The difference between EG and CG in physical exertion is shown in Figure 2A.

**Table 1:** Demographic characteristics and descriptive statistics on raw data (means and standard deviation [in parentheses]) of physical and perceived exertion, anticipation to run, and joy of running of participants in the experimental and control groups.

	**Experimental group**	**Control group**
	**(EG, *n* = 20)**	**(CG, *n* = 20)**
Boys, *n* (%)	20 (100)	20 (100)
Age, years, Mean (SD)	16.35 (0.49)	16.20 (0.41)
Body mass index, Mean (SD)	19.49 (2.69)	22.85 (4.48)
Physical activity, h/week, Mean (SD)	4.55 (3.51)	3.53 (2.62)
Physical exertion, beats per minute, Mean (SD)	168.20 (9.64)	150.78 (19.93)
Intensity, % of the 30 min running time, Mean (SD)		
Very light (50–60%)	1.85 (4.70)	5.32 (10.25)
Light (60–70%)	3.35 (6.40)	33.73 (31.44)
Moderate (70–80%)	14.35 (19.79)	36.11 (29.98)
Intense (80–90%)	77.55 (25.21)	12.37 (17.82)
Maximum (>90%)	2.85 (6.67)	12.37 (22.64)
Perceived exertion, Borg's RPE, Mean (SD)	13.00 (2.13)	10.63 (3.70)
Anticipation to run, Mean (SD)	2.20 (1.15)	2.21 (0.92)
Joy of running, Mean (SD)	3.20 (0.95)	2.63 (0.96)

**Figure 3:**
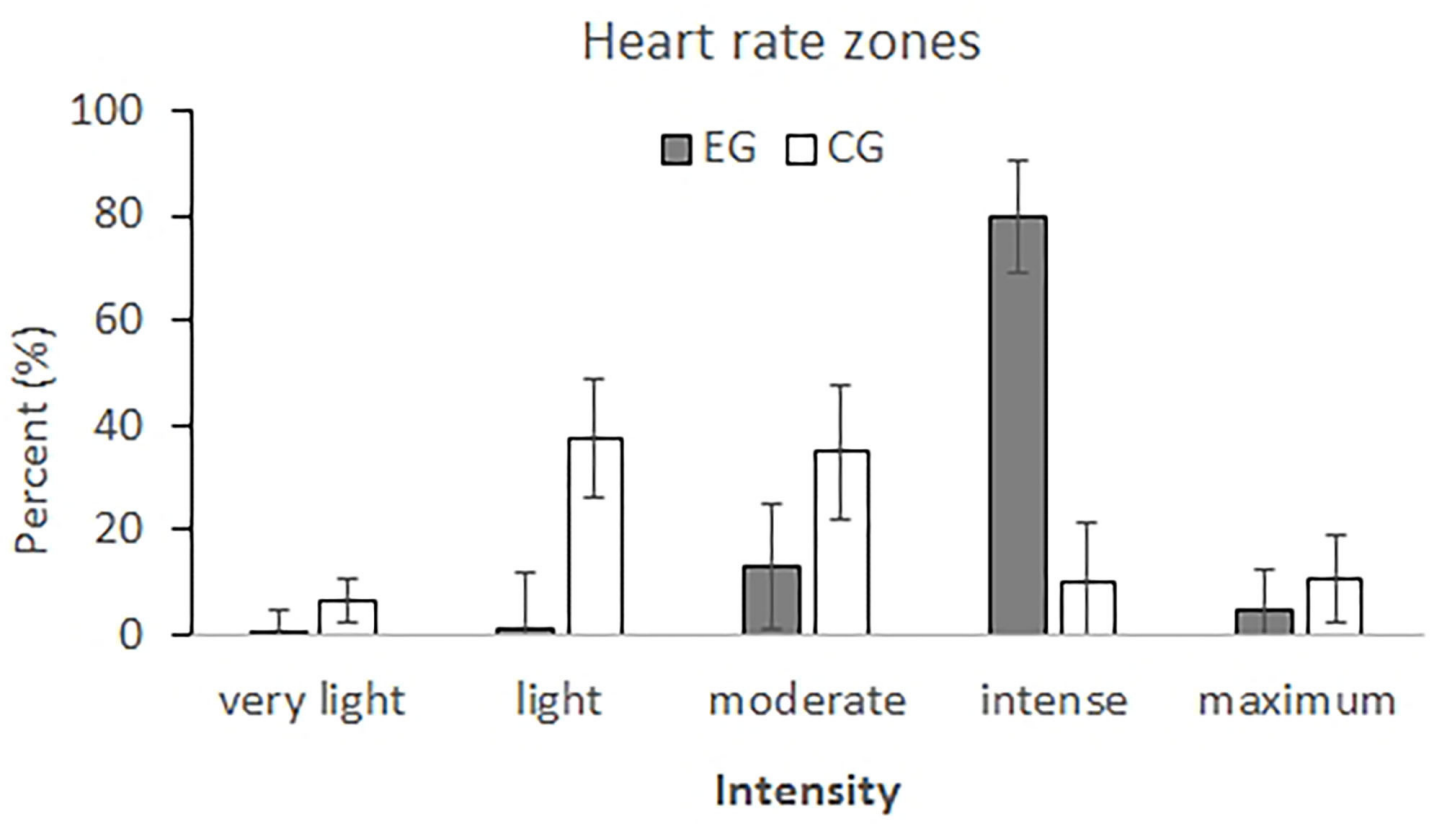
Time (in percent) students of the experimental (EG, gray bars) and control group (CG, white bars) were running at very light (50–60% of maximum heart rate), light (60–70%), moderate (70–80%), vigorous (80–90%), and maximum intensities (90–100%) during the 30 min run. Error bars indicate 95% CI.

Analysis of time (in percent) spend in the five Polar® heart rate zones revealed a group × heart rate zone interaction, *F*_(4,140)_ = 25.51, *p* < 0.001, η_*p*_^2^ = 0.42. *Post-hoc* analysis revealed that participants of the EG spend more time in heart zone 4 (*MD* = 69.29%, *95% CI* = [52.72, 85.85], *p* = 0.001), and less time in heart rate zone 2 (*MD* = −36.08%, *95% CI* = [−52.66, −19.50], *p* < 0.001) and heart rate zone 3 (*MD* = −21.88%, *95% CI* = [−40.41, −3.35], *p* = 0.02) than participants of the CG. The differences between EG and CG for the time spent in the five Polar® heart rate zones are presented in Figure 3.

### Perceived Exertion

Data analysis revealed a significant difference between groups, *F*_(1,35)_ = 5.77, *p* = 0.02, η_*p*_^2^ = 0.14, with a higher RPE in the EG (*M* = 13.22, *95% CI* = [11.78, 14.66]) as compared to the CG (*M* = 10.59, *95% CI* = [9.06, 12.12]) (adjusted Cohen's *d* = 0.74, *95% CI* = [0.06, 1.50]). Based on these means and 95% CIs of the mean, on average participants of the EG engaged in moderate to vigorous physical activity, while participants of the CG engaged in light to moderate physical activity (American College of Sports Medicine, 2014). The difference between groups in perceived exertion is shown in Figure 2B. Correlation analysis revealed that perceived exertion was positively related to physical exertion (*r* = 0.53, *p* = 0.001).

### Motivation

**Figure 4:**
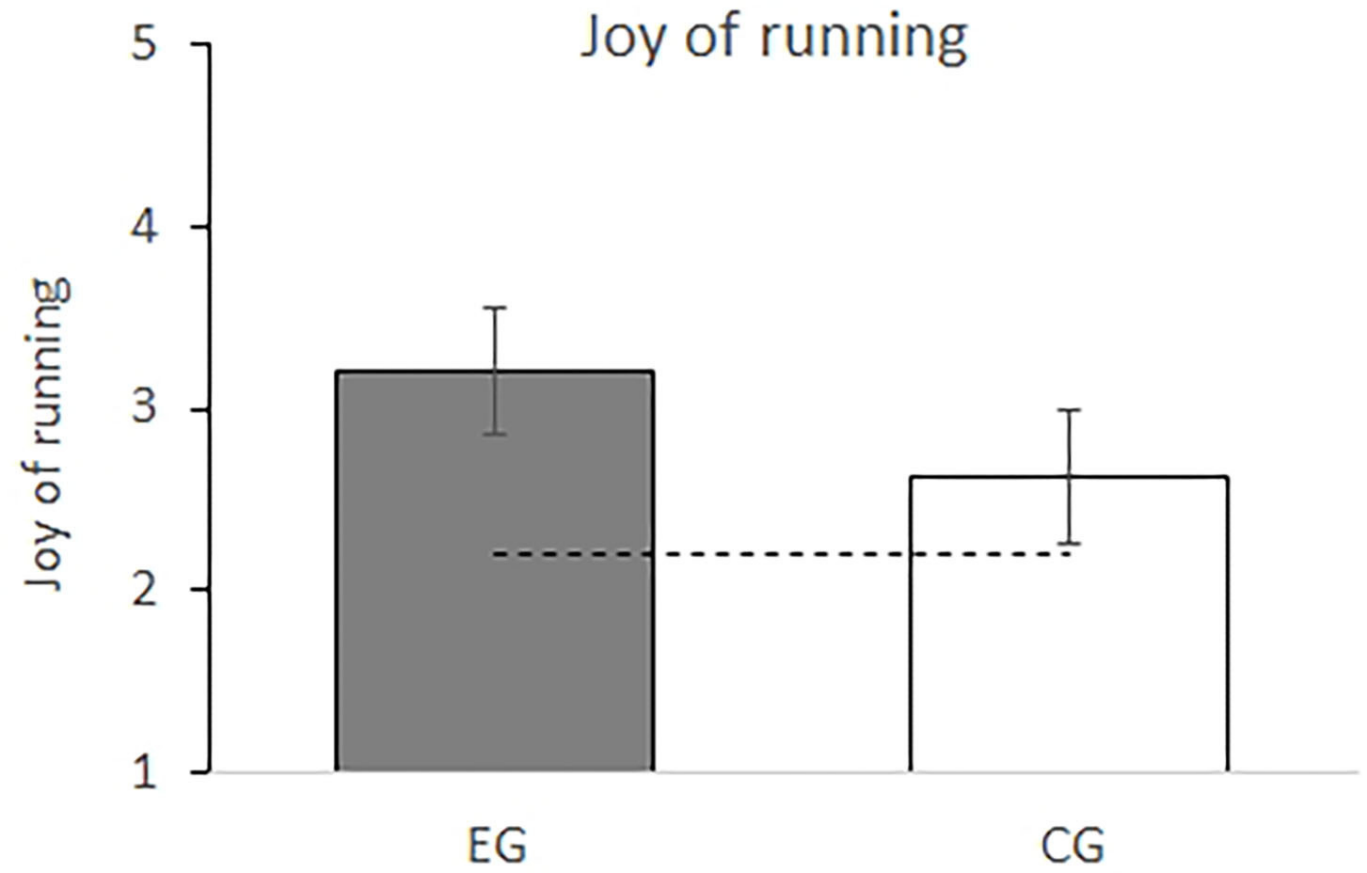
Self-report of how much students enjoyed the 30 min run in the experimental (EG, gray bar) and control groups (CG, white bar) with higher scores indicating greater enjoyment. Error bars indicate 95% CI. The dashed line indicates the average of participants' anticipation to run before any running took place (*M* = 2.21).

Analysis of participants' motivation revealed a significant difference between groups, *F*_(1,36)_ = 5.26, *p* = 0.03, η_*p*_^2^ = 0.13. Data analysis revealed that participants in the EG (*M* = 3.20, *95% CI* = [2.85, 3.56]) enjoyed running more than participants in the CG (*M* = 2.63, *95% CI* = [2.27, 2.99]) (adjusted Cohen's *d* = 0.69, *95% CI* = [0.03, 1.43]). Anticipation to run (i.e., motivation before any running) did not differ between groups (*MD* = −0.01, *95% CI* = [−0.69, 0.67]; *p* = 0.98). The difference in motivation between the two groups is shown in Figure 4.

## Discussion

The purpose of the present study was to examine whether the use of real-time, individualized heart rate feedback during PE has the potential to increase students' joy of running, which would be indicative of an increased autonomous motivation toward running. To achieve this, we had forty adolescents run for 30 min either with (EG) or without real-time, individualized heart rate feedback during PE and compared physical and perceived exertion as well as joy of running between the two groups. Our data revealed that participants in the EG enjoyed running more than participants in the CG despite a higher physical and perceived exertion.

In detail, we found that the level of physical exertion during the 30 min run was higher in the EG than in the CG. More specifically, students that were able to use the individualized, real-time heart rate feedback (EG) engaged in vigorous physical activity (163–178 bpm) at an average of about 83% of their maximum heart rate, while students without access to real-time heart rate feedback (CG) engaged in moderate physical activity (141–156 bpm) at an average of about 73% of their maximum heart rate. Participants in the CG spend more time in heart rate zones associated to light (37.4% of the running time) or moderate intensities (35.1% of the running time) as opposed to participants in the EG who spend most of their 30 min run in heart rate zone 4 (79.5% of the running time) which is associated to vigorous physical activity. Thus, it appears that the use of individualized, real-time heart rate feedback helped students to push themselves harder and run at higher intensities over the course of the 30 min run (i.e., vigorous instead of light to moderate physical activity).

Of particular importance, our data suggests that students tend to (willingly) underperform (i.e., to run at light to moderate intensities) if not being provided with real-time, individualized feedback of their running performance. Ratings of perceived exertion indicated that the lower intensities in the CG are rather an intentional choice of the students (e.g., a lack of motivation) than a perceived limitation of own resources (i.e., physical capacities) as these students seem to be well aware of the low intensities. Participants in the CG rated their perceived level of exertion after the run as very light to light, while participants in the EG rated it as somewhat hard to hard. Ratings of perceived exertion thus indicate that adolescents in our study were not willing to push themselves hard while running (i.e., lack of motivation), unless they received real-time, individualized heart rate feedback which apparently gave them a reason to push themselves harder (i.e., being motivated due to feedback of goal attainment). This is in line with research indicating that (digital) individualized feedback of performance, learning and goal attainment encourages students to put more effort into improving themselves and to keep them engaged with an activity (Alderman et al., 2006; Bice et al., 2016; Nation-Grainger, 2017). For example, Alderman et al. (2006) argued that (individualized) feedback of performance and learning (as opposed to outcome feedback) encourages students to test different solutions during skill acquisition (i.e., trial and error learning) and to put more effort into improving the skill. Bice et al. (2016) found that real-time, individualized activity tracking significantly increased participants' motivation toward physical activity. They argued that feedback and individual goal setting keeps participants engaged and motivated (i.e., provides a reason for them to perform).

Most important, participants in the EG enjoyed running more than those in the CG despite higher levels of physical and perceived exertion. Anticipation to run did not differ between both groups; on average neither group did really look forward to run. While that didn't change in the CG over the course of running, the EG reported a higher joy of running after the run as compared to their initial anticipation to run before the 30 min run. That means, running with real-time, individualized heart rate feedback apparently increased participants' motivation to run and to enjoy running at higher levels of exertion, which is in line with findings on the relation between physical activity and positive affect (Cameron et al., 2018; Ludwig and Rauch, 2018). In detail, students' may have experienced positive affects as they were in control of the situation (real-time adjustments possible) and felt competent (individualized feedback), which led to an increase in their effort to achieve their goals.

That said, the most intriguing finding of the present study was that students provided with real-time, individualized heart rate feedback enjoyed running more than those without such feedback, despite the fact that they pushed themselves harder during the 30-min run (as evidenced by significantly higher levels of physical and perceived exertion in the EG). Thus, real-time, individualized heart rate feedback apparently enhanced students' motivation toward running (i.e., toward a more internal form of regulation). Based on SDT (Deci and Ryan, 2000; Ryan and Deci, 2000, 2007, 2017), autonomous forms of motivation can be achieved by satisfaction of the three psychological needs competence, autonomy and relatedness. With regard to motivation toward PE and physical activity, especially the needs of competence and autonomy have been shown to be positively associated to autonomous forms of motivation (Standage et al., 2003, 2006; Ntoumanis, 2005; Standage and Gillison, 2007; Hashim et al., 2008; Taylor et al., 2010; Gråstén et al., 2012; Ryan and Deci, 2017). In a study by Ntoumanis (2001), students reported that individualized feedback made PE more interesting, they enjoyed it more and they wanted to improve more. Kalaja et al. (2009) found that adjusting task demands during PE to individual levels of exercise highly contributes to the feeling of perceived competence. Similarly, Alderman et al. (2006) argued that (individualized) feedback of performance and learning encourages students to put more effort into improving the skill as they feel more in control of their own performance. In that regard, it is very likely that the individualized activity feedback (i.e., based on each participant's level of exercise) in the EG helped participants to feel more competent (Ntoumanis, 2001; Alderman et al., 2006; Bice et al., 2016) by being able to effectively adjust own behavior (i.e., running speed) to achieve an individualized (i.e., realistic) goal. Moreover, the possibility to actively adjust running speed based on self-monitoring of heart rates at all times (i.e., having control over their own performance in terms of effort, exhaustion, relaxation and goal attainment) probably supported the need for autonomy in the EG. Although relatedness appears not as important as autonomy and competence for enhancing and maintaining autonomous forms of motivation (Deci and Ryan, 2000; Standage and Gillison, 2007), the immediate visibility (and possibility to compare) of one's performance to others could have added an element of competition to the urge to achieve the predefined goal (for the group), i.e., supporting externally driven forms of regulation. That means, the “group pressure” (i.e., a kind of relatedness) might have helped those participants that could not build up any internal desire to run (i.e., students with low autonomous motivation toward PE).

There are some limitations to the current study, which may inform future directions in this line of research. First, we only tested adolescents of 16–17 years of age, an age group already known for a low motivation toward PE (Ntoumanis, 2001; Ntoumanis et al., 2004) and physical activity in general (Knuth and Hallal, 2009; Hallal et al., 2012; Van Hecke et al., 2016). While from a practical perspective this is surely the age group that needs motivation enhancing methods in PE the most, it is also the group for which largest motivation and performance changes can be expected as there is probably more room for improvements than in any other age group. In that regard, it might be worthwhile to study other age groups and see how the positive effects of immediate activity feedback change with increasing age and different forms of motivation toward PE and physical activity in general. Second, in order to be able to easily implement heart rate monitoring and feedback in regular PE classes a simplified approach to determine the individual maximum heart rate (220 bpm minus age; Fox et al., 1971) was chosen in the present study. While this makes sense from a practical point of view (i.e., feasibility in the school-setting), it does not sufficiently consider individual differences in maximum heart rate that have been found to greatly depend on individual physiology and environmental factors (Zhu et al., 2010; cf. Sarzynski et al., 2013). However, with the goal in mind to implement heart rate monitoring and feedback in the school-setting, it is probably the best compromise to start with the Fox formula (Fox et al., 1971), as it appears good enough to have a positive impact on students motivation toward PE, and adjust students' maximum heart rate measures based on experience when required (e.g., when observing marked differences between estimates and indicators of effort and exhaustion). However, given the nature of the measures (also regarding self-report data on anticipation and joy of running), the present results should be understood in context and interpreted with caution. Third, it's almost impossible to tease apart the social component of the intervention from the heart rate feedback. By nature of the intervention, there was more interaction with the instructor in the EG than in the CG even though the instructor was asked to limit the interaction to the necessary information. This may indeed have facilitated the hypothesized response (i.e., increase in autonomy and physical effort) in the EG. However, even if immediate heart rate feedback only helped increase students' motivation toward PE due to the increased social interaction, its implementation in regular PE settings would still be beneficial and desired. Finally, participants in the present study were only confronted with heart rate monitoring and feedback on a single occasion. That means that based on the present data it is impossible to draw meaningful conclusions on whether the use of individualized, real-time heart rate feedback during PE classes affects students' motivation toward running, PE or physical activity in general, or in a long term. It could well be that the positive effects wear off the longer the method is used or that the positive effects for running during PE do not generalize to other forms of physical activity during PE or in general. McManus et al. (2008) for example found that heart rate feedback led to modest increases in daily time children spent above 140 bpm and percentage of time spent being vigorously active, however, these changes in behavior were not lasting when feedback was removed. In that regard, future studies should look into the long term and transfer effects of autonomy and competence supportive methods during PE, and real-time, individualized heart rate feedback in particular. Moreover, more studies are needed that inform practitioners (i.e., PE teachers, coaches, or instructors) about how such methods are applied in specific situations to guarantee maximum gain. For example, while it appears conclusive that introducing real-time, individualized activity feedback for the first time has immediate positive effects on motivation, future studies should tackle the question on how, when or how often such methods should be implemented to preserve or enlarge these positive effects.

Limitations notwithstanding, findings of the present study demonstrate that the use of immediate, individualized activity feedback during endurance training (i.e., running) in PE has the potential to increase students' joy of running (i.e., motivation to run) and effort during PE. This is of great importance since enjoyment of PE has been found to have a positive impact on children's extracurricular physical activity (Dishman et al., 2005; Cairney et al., 2007; Cox et al., 2008). That means, understanding the benefits and experiencing the satisfaction of physical activity will ultimately help children to develop an autonomous (i.e., intrinsic) motivation toward physical activity in general.

In that regard, real-time, individualized heart rate feedback or biofeedback in general (i.e., direct or indirect feedback of a physiological process given without interrupting the exercise) should be implemented in regular PE classes systematically and repeatedly (whenever required to increase autonomous forms of motivation toward physical activity during PE) to create a controllable (autonomy) and attainable (competence) situation that allows *all* students to actively adjust (self-endorsed) their own behavior to achieve appealing and realistic goals. At the very least, the visibility of own (under)performance to self and others should help students to push harder while exercising. As especially heart rate sensors are becoming smaller, better, more reliable, and cheaper almost on a daily basis, and they are easy to use even in larger groups in the school setting, it is probably one of the technical gadgets that has the potential to provide for sustainable improvements of PE classes and become a regular measuring and feedback tool in the majority of PE classes worldwide in the future.

## Data Availability Statement

The raw data supporting the conclusions of this article will be made available by the authors, without undue reservation.

## Ethics Statement

Ethical review and approval was not required for the study on human participants in accordance with the local legislation and institutional requirements. Written informed consent to participate in this study was provided by the participants' legal guardian/next of kin.

## Author Contributions

TS and RG developed the study protocol. RG was responsible for data collection. Both authors equally contributed to the analysis and interpretation of the results, and to drafting the article. Both authors approved the final version.

## Conflict of Interest

The authors declare that the research was conducted in the absence of any commercial or financial relationships that could be construed as a potential conflict of interest.

## Abbreviations

MPA: moderate physical activity
VPA: vigorous physical activity
bpm: beats per minute
PE: physical education
SDT: self-determination theory
EG: experimental group
CG: control group
BMI: body-mass index
RPE: rating of perceived exertion.
