# Supplementary material for: Psychophysiological Benefits of Real-Time Heart Rate Feedback in Physical Education
Source: Front Psychol. 2021 Mar 16;12:651065. doi: 10.3389/fpsyg.2021.651065 (PMC8009282; doi:10.3389/fpsyg.2021.651065)
Supplement: Supplementary file 1 [file Data_Sheet_1.docx]

**Supplementary material to**

**“Psychophysiological benefits of real-time heart rate feedback in physical education”**

Stöckel, Tino & Grimm, Robert

**S1. Student self-report on anticipation to run (before running)**

**S2. Student self-report on joy of running (after running)**

**S3. Student self-report on perceived exertion (after running)**

**S1. Anticipation to run:** Before any running the student were asked to self-report whether they are looking forward to the upcoming 30 minute run. The students were handed a piece of paper with the following information and question on it, and were asked to make a cross underneath the respective smiley that best represented their current anticipation to run on an individual basis.

Name: ____________________________
Sensor number: ____

Date and time: ____________________ ____________________

Class: ____

Please report whether you agree with the following: **I AM LOOKING FORWARD TO RUN TODAY!**


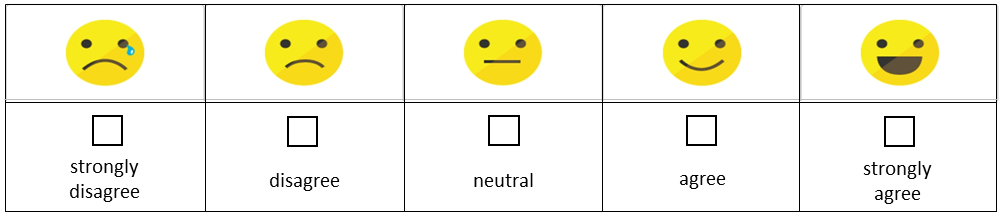


**S2. Joy of running:** Directly after the 30 minute run, the student were asked to self-report whether they enjoyed the 30 minute run. Again the students were handed a new piece of paper with the following information and question on it, and were asked to make a cross underneath the respective smiley that best represented their enjoyment during running on an individual basis.

Name: ____________________________
Sensor number: ____

Date and time: ____________________ ____________________

Class: ____

Please report whether you agree with the following: **I ENJOYED RUNNING TODAY!**


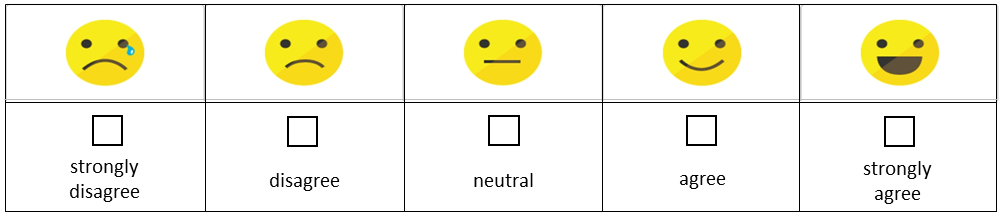


**S3. Rating of Perceived Exertion (Borg RPE scale):** Directly after the 30 minute run and together with reporting the joy of running, the student were asked to self-report how exhausted they felt during the 30 minute run. On an individual basis, they had to make a cross at the description that best matched their level of exertion during running.

Please report your level of exertion during the 30 minute run – how hard you had to push yourself?

Make a cross at the respective number/description!

Name: ____________________________
Sensor number: ____

| cross | Borg Scale |
| --- | --- |
|  | **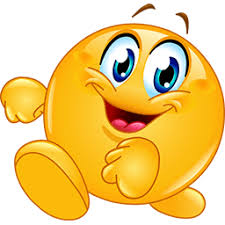6 No exertion** |
|  | **7 Extremely light** |
|  | **8** |
|  | **9 Very light** |
|  | **10** |
|  | **11 Light** |
|  | **12** |
|  | **13 Somewhat hard** |
|  | **14** |
|  | **15 Hard** |
|  | **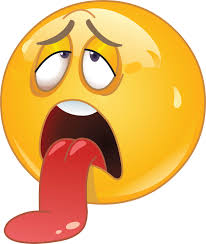16** |
|  | **17 Very hard** |
|  | **18** |
|  | **19 Extremely hard** |
|  | **20 Maximal exertion** |
